# Ultrasound‐Defined Sarcopenia Independently Predicts Acute Decompensation in Advanced Chronic Liver Disease

**DOI:** 10.1002/jcsm.13630

**Published:** 2024-11-11

**Authors:** Juliana Gödiker, Lea Schwind, Torid Jacob, Nina Böhling, Sara Noemi Reinartz Groba, Markus Kimmann, Jörn Arne Meier, Kai‐Henrik Peiffer, Jonel Trebicka, Johannes Chang, Michael Praktiknjo

**Affiliations:** ^1^ Department of Internal Medicine B University Hospital Münster Münster Germany; ^2^ Department of Internal Medicine I University Hospital Bonn Bonn Germany; ^3^ Center for Cirrhosis and Portal Hypertension Bonn (CCB) University Hospital Bonn Bonn Germany

**Keywords:** ACLF, acute decompensation, chronic liver disease, cirrhosis, malnutrition, portal hypertension, sarcopenia, skeletal muscle index, ultrasound

## Abstract

**Background:**

It has been shown that in patients with liver cirrhosis, sarcopenia is a predictor of acute decompensation (AD), acute‐on‐chronic liver failure (ACLF) and death. However, computer tomography (CT), as a suggested standard method for diagnosing sarcopenia, is resource intensive and involves radiation exposure. Therefore, in this study, we evaluate the muscle thickness of quadriceps femoris measured by ultrasound (US) as a prognostic parameter for AD and all‐cause mortality in chronic liver disease.

**Methods:**

Sixty‐three patients with chronic liver disease and signs of portal hypertension were analysed in this prospective monocentric study for the occurrence of acute decompensation such as hepatic encephalopathy, ascites, haemorrhage and liver‐related death within 1 year. We assessed muscle thickness at three different heights in terms of suitability as a predictor.

**Results:**

Among all 63 patients, 15 patients experienced acute decompensation, and 9 patients died due to liver‐related death. We found the upper third of the muscle, measured without applying pressure with the transducer, to be the most significant for predicting AD/ACLF [AUC 0.739 (confidence interval (CI) 0.604–0.874, *p* = 0.006]. A cut‐off value of US‐defined muscle thickness standardized per height for identifying sarcopenia was determined (1.83 cm/m). Patients with US‐defined sarcopenia showed significantly higher rates of AD (38.9% vs. 3.7%, *p* = 0.001) and all‐over 1‐year mortality (27.8% vs. 3.7%, *p* = 0.013). The mean AD free survival time is 8.3 months (95% CI 6.6–9.9) for sarcopenic patients and 11.8 months (95% CI 11.0–12.6) for the non‐sarcopenic cohorts. Corresponding CT analysis displayed similar results for AD free survival for both groups (40% AD rate in the sarcopenic group vs. 7% AD rate in the non‐sarcopenic group, *p* = 0.001). The risk for AD was significantly higher in the sarcopenic cohort compared with those without sarcopenia in both US and CT (US: HR 16.6; *p* = 0.009; 95% CI 2.0–136.0; CT: HR 8.7; *p* = 0.017; 95% CI 1.5–51.0). CT and US displayed a moderate agreement (*p* = 0.006; κ = 0.379).

**Conclusions:**

Sarcopenia classification based on US measurements is shown to be an independent predictor of AD occurrence within 1 year. This pilot study is the first to suggest that screening for sarcopenia by ultrasonography may be useful for risk assessment in patients with chronic liver disease and signs of portal hypertension.

**Abbreviations**
ACLF: acute‐on‐chronic liver failure
AD: acute decompensation
BIA: bioelectrical impedance analysis
CSPH: clinical significant portal hypertension
CT: computed tomography
EASL: European Association for the Study of the Liver
HCC: hepatocellular carcinoma
HE: hepatic encephalopathy
HGS: hand grip strength
MELD: model for end‐stage liver disease
MRI: magnetic resonance imaging
NCPH: non‐cirrhotic portal hypertension
TIPS: transjugular intrahepatic portosystemic shunt
US: ultrasound

## 1 Introduction

Sarcopenia, defined by the European Working Group on Sarcopenia in Older People (EWGSOP) as the presence of low muscle strength plus low muscle quantity or quality [1], is a common and often underestimated complication in patients with liver cirrhosis. Despite the revised EWGSOP2 definition prioritizing muscle strength as the primary parameter for sarcopenia, this study will utilize muscle quantity as the diagnostic criterion. This approach is adopted due to the observed underdiagnosis of sarcopenia when employing the revised EWGSOP2 criteria [2]. Its prevalence in patients with cirrhosis is estimated to be 30%–70% [3, 4, 5].

Diagnosing sarcopenia in patients with liver disease is highly important, as it is shown to have a crucial impact on morbidity and mortality both pre‐ and post‐liver transplantation [5, 6].

Indeed, low skeletal muscle mass, diagnosed mostly by CT, has been shown to be an independent risk factor for the development of overt hepatic encephalopathy (HE), infections, ascites and acute‐on‐chronic‐liver failure (ACLF) and influences the clinical outcome after transjugular intrahepatic portosystemic shunt (TIPS) implantation [5, 6, 7, 8, 9, 10].

Pathophysiologically, several mechanisms contribute to muscle wasting in cirrhotic patients. Besides malnutrition due to loss of appetite and physical inactivity, decreased testosterone and growth hormone levels, hyperammonia itself is believed to be a mediator of the liver‐muscle axis by impairment of protein synthesis and increased autophagy and mitochondrial oxidative dysfunction [11, 12, 13, 14, 15].

The European Association for the Study of the Liver (EASL) proposed several methods to assess sarcopenia. Alternatively, mid‐arm muscle circumference, whole body dual‐energy X‐ray absorptiometry, tetrapolar bioelectrical impedance analysis (BIA), handgrip strength (HGS) and magnetic resonance imaging (MRI) are proposed as various techniques to evaluate sarcopenia, with each having its own limitations such as high costs or complex technical implementation. However, evaluation of total cross‐sectional area (cm^2^) of abdominal skeletal muscles at L3 [skeletal muscle index (SMI) normalized to height] by computer tomographic (CT) imaging is currently suggested as standard approach to assess sarcopenia by the EASL guidelines [16].

Importantly, ultrasound (US) examinations are commonly performed in these patients. In most current guidelines, US is recommended every 6 months for HCC screening in high‐risk patients [17]. Moreover, patients with ascites are often followed up by US at even higher frequencies. In geriatrics and other fields, US has been explored for the assessment of skeletal muscle mass with promising results [18, 19, 20]. In contrast to CT and other methods, US is a commonly available cost‐ and time‐effective technique without radiation exposure, which can be performed as a point‐of‐care diagnostic at bedside.

In the last few years, several studies demonstrated the relation between CT‐acquired SMI and mortality and morbidity in cirrhotic patients, but there is only limited data about the association of US‐defined sarcopenia with AD and mortality in chronic liver disease. Our aim was to investigate whether US is a reliable technique for the prediction of sarcopenia and acute decompensation and mortality in these patients.

We hypothesized that low thigh muscle thickness diagnosed by US would be associated with a higher rate of liver‐related complications within 1 year and may be a prognostic marker.

## 2 Methods

### 2.1 Study Population

In this monocentric observational prospective study, we included patients with advanced chronic liver disease admitted to hospital between 2017 and 2019 without option for TIPS. Exclusion criteria were non‐liver‐related severe disease with a life expectancy of less than 1 year and incomplete US measurements or TIPS implantation as it changes the natural disease history (Figure S1). The follow up period was 1 year, and primary outcome were episodes of acute decompensation (AD). The first AD was considered censoring event. AD was defined as ascites requiring treatment (paracentesis and/or hospital admission), development of overt hepatic encephalopathy (grade II‐IV) and gastro‐eosophageal variceal haemorrhage [21]. Secondary outcome was liver‐related death due to ACLF, which was defined as described by Moreau et al. in the CANONIC study [22].

### 2.2 US‐Measurements of Muscle Thickness

The transversal muscle thickness of the quadriceps femoris was measured at three different locations (mid, upper and lower third) using a commercially available ultrasound system (Supersonic Aixplorer, Hologic, Aix‐en‐Provence, France) with a 2–10 MHz transducer. US measurements were performed by DEGUM (German Association for Ultrasound in Medicine) certified hepatologists within 48 h after admission. The transversal muscle thickness between the femoral bone and the subcutaneous adipose tissue was measured completely, and three measurements in centimetres were performed with and without applying pressure respectively. Mean values were calculated and the muscle thickness was—analogous to the CT‐defined skeletal muscle index (SMI)—standardized to the patient's height to calculate US‐defined skeletal muscle index (US‐SMI).

### 2.3 CT Measurement of Muscle Thickness

At baseline, 43 patients underwent a routine diagnostic abdominal CT (iCT, Philips Healthcare, Amsterdam, Netherlands) for routine clinical evaluation in supine position with iodinated contrast being administered as described previously [8]. An attenuation range from −30 to 150 Hounsfield units (HU) was used to identify muscles. The skeletal muscle index, as defined by the EASL, is assessed by evaluating the cross‐sectional area of skeletal muscle at the L3 vertebra. The SMI is calculated by normalizing the muscle mass to the patient's height. Thresholds of 50 cm^2^/m^2^ for men and 39 cm^2^/m^2^ for women were used.

### 2.4 Statistical Analysis

All variables were analysed by descriptive statistics. Non‐parametric tests were used to determine statistical significance between groups. Mann–Whitney *U* is used to analyse the differences between the various ultrasound sites. Receiver operating characteristic (ROC) analysis was performed to determine optimal cut‐off values for muscle thickness measured by US. Optimal threshold was determined by Youden index. Spearman test and Cohen's kappa were used to compare association and agreement between US‐ and CT‐defined muscle thickness. Missing data were excluded, and lost to follow up was censored. *p* < 0.05 was considered statistically significant. Kaplan–Meier curve with log‐rank test was used to assess AD free survival stratified by muscle thickness. Separate Kaplan–Meier curves are plotted for both types of measurement (US and CT), showing the probability of acute decompensation/AD ACLF over time. The curves were compared by log‐rank test to see if there is statistical difference between both measurement in predicting the outcome. Logistic regression was used for univariate followed by multivariate risk factor analyses. Multivariate analysis was performed separately with MELD and Child–Pugh score to avoid collinearity. Continuous variables are reported as median (range), if not otherwise specified, and categorical variables are reported as absolute cases or percentages. *p* < 0.05 was considered statistically significant. For all statistical data analysis, SPSS (version 26) was utilized.

## 3 Results

### 3.1 Patient Characteristics

In total, 63 patients were included in the study. The general characteristics are shown in Table 1. Most of the patients were male (45 patients, 69%) and the median age at baseline was 61 (23–87) years. Most of the patients (44.4%) had alcohol‐related cirrhosis, 3.2% had viral hepatitis, 20.7% had NAFLD‐related cirrhosis and 31.7% of patients had chronic liver disease of other aetiology such as autoimmune liver disease. The median MELD was 11 and the median Child–Pugh score was 7. Overall, 15 patients (23.8%) developed AD within 1 year, with several patients also experiencing multiple episodes of AD (total of 23 events). Nine patients died due to fatal ACLF.

**TABLE 1:** Patient characteristics.

Parameter	Baseline (*n* = 63) (range)
Clinical	
Age (in years)	61 (23–87)
Sex (male/female)	45/18 (71/29%)
Aetiology of liver disease	
Alcohol‐induced liver cirrhosis	28 (44.4%)
Viral hepatitis	2 (3.2%)
NAFLD‐related cirrhosis	13 (20.7%)
Other aetiology	20 (31.7%)
Height (cm)	174 (153–201)
Weight (kg)	82 (43–130)
BMI (kg/m^2^)	25.9 (16.8–41.4)
Decompensation status at BL (decompensated/compensated)	26/37 (41.3%/58.7%)
Ascites at BL	20 (31.7%)
Variceal bleeding at BL	6 (9.5%)
Scores	
MELD	11 (6–39)
Child–Pugh	7 (5–11)
Laboratory	
Sodium (mmol/L)	139 (124–147)
Creatinine (mg/dL)	0.9 (0.4–6.65)
Bilirubin (mg/dL)	1.1 (1–9.8)
Urea (mg/dL)	139 (9.5–230)
gGT (U/L)	92 (20–980)
AST (U/L)	42 (18–675)
ALT (U/L)	28 (10–299)
INR	1.2 (1.0–3.0)
Platelets (G/L)	143 (25–743)
Albumin (g/L)	34 (17.6–52.8)
Outcome	
AD	15 (23.8%)
Ascites	15 (23.8%)
HE	5 (8%)
Variceal bleeding	3 (5%)
Liver related death	9 (14%)
Mortality at 1‐year follow‐up	11 (17%)
Reason for death (ACLF/other)	9/2 (82/18%)

Abbreviations: ACLF, acute‐on‐chronic liver failure; AD, acute decompensation; ALT, alanine transaminase; AST, aspartate transaminase; BMI, body mass index; gGT, gamma‐glutamyltransferase; HE, hepatic encephalopathy; INR, international normalized ratio; MELD, model for end‐stage liver disease.

Subgroups of the cohort due to their decompensation status at baseline were defined; 41.3% of the patients were decompensated during assessment such as ascites (31.7%) and variceal bleeding (9.5%). 58.7% of the patients were in a stable phase without acute decompensation at the time of evaluation. This subgroup is referred to as ‘compensated’ (Table 1).

### 3.2 US‐Based Sarcopenia Classification

The measured muscle thickness values, without pressure being applied with the transducer, were more significantly associated with the development of AD episodes. Among the different measurement sites, the measurements at the proximal third of quadriceps femoris applied without pressure were the most significant in predicting the outcome [Mann–Whitney *U*, *p* = 0.009; ANOVA, *p* = 0.007 (left) and *p* = 0.006 (right)] (Table S1). The middle third is also significantly associated with AD both left and right (ANOVA left *p* = 0.031, right *p* = 0.028). The significance was found to be greater in all measurements when the examiners' pressure was absent, which may be attributed to the varying degrees of strength applied. To simplify the measurements, we continued our analyses with the measured values of the left side. There was no significant difference between the left and right muscle thickness parameters.

Thus, we decided to use this parameter (left, proximal third, no pressure) standardized for height in cm/m and performed ROC analyses with the occurrence of AD within 1 year as end point.

The ROC analysis for the development of AD showed an AUC of 0.739 (CI 0.604–0.874, *p* = 0.006). We determined cut‐off values of 1.8 cm/m. Patients below this threshold were defined as sarcopenic. Due to the limited numbers of female patients, no sex‐specific measurements were performed.

### 3.3 US‐Defined Sarcopenia and AD Development

**FIGURE 1:**
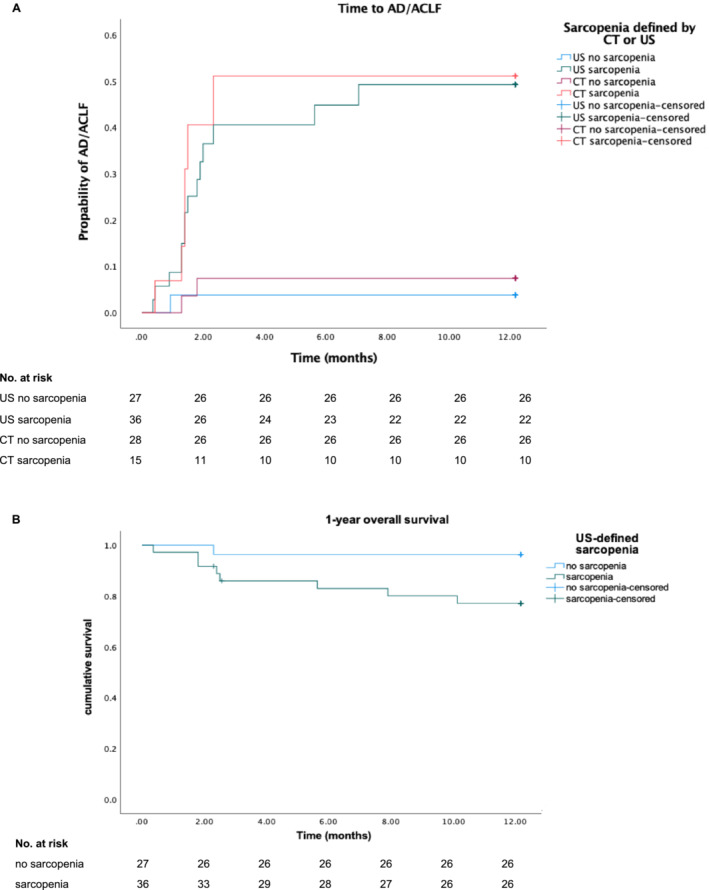
(A) Time‐to‐event curve for occurrence of AD/ACLF in patients with chronic liver disease with or without sarcopenia as determined by ultrasound measurement (*p* = 0.003) or CT measurement (*p* = 0.05). (B) Kaplan–Meier curve for all‐cause survival of patients with and without sarcopenia defined by US (*p* = 0.038).

Among all 63 patients, 15 patients met the primary endpoint, and 9 patients met the secondary endpoint. Overall, 36 (57.1%) were defined as sarcopenic by ultrasound. Sarcopenic patients showed significantly increased rates of AD development compared with the non‐sarcopenic patients as shown in the time‐to‐event curve (38.9% vs. 3.7%, *p* = 0.001) (Figure 1A). A Kaplan–Meier survival curve for 1‐year mortality showed a significantly increased mortality in sarcopenic patients using the cut‐offs as determined above (*p* = 0.001) (Figure 1B). The mean survival time was 9.2 months (95% CI 7.7–10.8) in the sarcopenic cohort compared with 11.8 months (95% CI 11.0–12.6) in the non‐sarcopenic cohort. The AD free survival within 1 year was significantly higher in the non‐sarcopenic group compared with the sarcopenic group [mean overall AD free survival 11.8 months (95% CI 11.0–12.6) vs. 8.3 months (95% CI 6.6–9.9)] (Figure 1A).

As expected, in addition to the distinctly different outcome parameters, the patient groups differed significantly in age [63 (41–87) vs. 52 (23–73 years), *p* = 0.001]. Noticeable is a significant difference in aetiology (*p* = 0.003) with significantly more alcohol‐induced liver cirrhosis in the sarcopenic group (61% vs. 22%). Besides, a significant difference in Child–Pugh [7 (5–9) vs. 5 (5–11), *p* = 0.038] and MELD score [14 (6–39) vs. 10 (6–20), *p* = 0.005] was shown (Table 2). Moreover, bilirubin and urea were significantly higher in sarcopenic patients. There were no significant differences in other laboratory results.

**TABLE 2:** General characteristics of patients with and without sarcopenia defined by ultrasound.

Parameter	Sarcopenia (*n* = 36)	No sarcopenia (*n* = 27)	*p* *
Clinical			
Age (in years) at baseline	63 (41–87)	52 (23–73)	**0.001**
Sex (male/female)	27/9 (75/25%)	18/9 (66.7/33.3%)	0.472
Aetiology of liver disease (alcohol/others)	22/14 (61/39%)	6/21 (22/78%)	0.003
Height (in cm)	174 (157–190)	174 (153–201)	0.884
Weight (in kg)	79.8 (54–130)	81.4 (43–115)	0.486
BMI (kg/m^2^)	26.3 (16.8–41.4)	26.6 (17.9–35.6)	0.585
Decompensation status at BL (decompensated/compensated)	19/17 (53/47%)	7/20 (26/74%)	0.034
Scores			
MELD	14 (6–39)	10 (6–20)	0.005
Child–Pugh score	7 (5–9)	5 (5–11)	**0.001**
Laboratory			
Bilirubin (mg/dL)	1.5 (0.2–9.8)	0.9 (0.3–5.6)	0.043
ALT (U/L)	29 (10–171)	25 (15–299)	0.053
Platelets (G/L)	134 (25–743)	164 (50–577)	0.305
Albumin (g/L)	31 (17.6–51.1)	42 (21.2–52.8)	**0.001**
Outcome			
AD	14 (38.9%)	1 (3.7%)	**0.001**
Ascites	10 (27.8%)	5 (18.5%)	0.397
HE	5 (13.9%)	0 (0%)	0.045
Variceal bleeding	3 (8.3%)	0 (0%)	0.127
Liver related death	6 (23%)	3 (8%)	0.039
Mortality at 1‐year	10 (27.8%)	1 (3.7%)	0.013
Reason for death (ACLF/other)	6/4 (60/40%)	3/1 (75/25%)	

*Note:* Data in bold indicate statistically significant.

Abbreviations: ACLF, acute‐on‐chronic liver failure; AD, acute decompensation; ALT, alanine transaminase; AST, aspartate transaminase; BMI, body mass index; gGT, gamma‐glutamyltransferase; HE, hepatic encephalopathy; INR, international normalized ratio; MELD, model for end‐stage liver disease.

* Patient groups were compared by Mann–Whitney *U* test and chi‐square test. Significance level is adjusted to p ≤ 0.0027 by Bonferroni correction to correct for multiple testing.

In the subgroup of compensated patients at baseline, 45.9% were identified as sarcopenic by ultrasound. Among these patients, 47.1% experienced decompensation within 1 year. It is noteworthy that in this subgroup of primary compensated patients, all patients who subsequently decompensated were identified as sarcopenic prior to this assessment by ultrasound (100%). In both subgroups, the number of decompensation events was nearly identical (21.6% in the non‐decompensated cohort vs. 26.9% in the decompensated cohort) (Table S2A,B). Kaplan–Meier curve demonstrated a significant association between acute decompensation and ultrasound‐defined sarcopenia even in patients with compensated liver disease at baseline (*p* = 0.001) (Figure S1A).

We performed univariate and multivariate logistic regression analyses. In univariate logistic regression, we found Child–Pugh score, MELD, international normalized ratio (INR), bilirubin and US‐defined sarcopenia to be dependent risk factors (Table 3). Moreover, US‐defined sarcopenia was confirmed as an independent predictor of the development of AD within 1 year in both multivariate analysis with MELD score [hazard ratio (HR) 9.2 *p* = 0.045] and multivariate analysis with Child–Pugh score (HR 12.6 *p* = 0.028) (Table 4).

**TABLE 3:** Univariate Cox regression analyses for AD within 1 year (*n* = 63).

Parameters	Univariate analysis		
	*p*	HR	95% CI
Age at baseline	0.056		
BMI at baseline	0.079		
Ultrasound defined sarcopenia	**0.009**	16.6	2.01–136.02
CT‐defined sarcopenia	**0.017**	8.7	1.5–51.0
Child–Pugh score	**0.003**	2.1	1.29–3.41
MELD	**0.002**	1.2	1.07–1.36
Aetiology	0.291		
INR at baseline	**0.035**	7.3	1.15–46.67
Bilirubin at baseline (mg/dL)	**0.009**	1.6	1.12–2.22
Creatinine at baseline (mg/dL)	0.056		
Sodium at baseline (mmol/L)	0.661		

*Note:* Data in bold indicate statistically significant.

Abbreviations: CI, confidence interval; HR, hazard ratio; INR, international normalized ratio; MELD, model of end stage liver disease.

**TABLE 4:** Multivariate Cox regression analyses for AD within 1 year of US‐ and CT‐defined sarcopenia with Child–Pugh and MELD score (*n* = 63).

	Multivariate analysis of US and Child–Pugh			Multivariate analysis of US and MELD			Multivariate analysis of CT and Child–Pugh			Multivariate analysis of CT and MELD		
	*p*	HR	95% CI	*p*	HR	95% CI	*p*	HR	95% CI	*p*	HR	95% CI
US defined sarcopenia	0.03	12.6	1.3–121.7	0.05	9.2	1.1–80.6						
CT‐defined sarcopenia							0.04	7.8	1.1–56.4	0.06	6.6	0.9–46.7
Child–Pugh score	0.02	1.9	1.2–3.3				0.03	3.1	1.1–8.4			
MELD				0.02	1.2	1.0–1.3				0.03	1.2	1.0–1.5

Abbreviations: CI, confidence interval; HR, hazard ratio; MELD, model of end stage liver disease.

The negative predictive value of non‐sarcopenic patients to remain stable was 96%. Positive predictive value of US‐defined sarcopenia was 39% (Table S3).

### 3.4 CT‐Defined Sarcopenia and AD

Besides US evaluation, in 43 patients, a corresponding CT measurement was performed to assess skeletal muscle index and identify CT‐defined sarcopenia within 6 weeks. For defining sarcopenia by CT, we used the thresholds proposed by the EASL (50 cm^2^/m^2^ for men and 39 cm^2^/m^2^ for women). In our cohort, 15 patients (35%) were identified as sarcopenic, which is consistent with the general prevalence of sarcopenia in cirrhosis in previous studies [5, 23]. In this subset, eight patients developed acute decompensation (19%).

In the sarcopenic group, 6 of 15 patients (positive predictive value = 40%) decompensated within 1 year, whereas in the non‐sarcopenic group, 2 patients were seen with an AD (7%). The negative predictive value is 93%.

As shown in the time‐to‐event curve in Figure 1A, the incidence of acute decompensation and ACLF increased significantly in the sarcopenic group defined by CT (*p* = 0.005). The mean AD free survival time in the CT defined‐sarcopenic group is in mean 7.6 months (r = 1.4; 95% CI 4.8–10.4) (Figure 1A).

**FIGURE 2:**
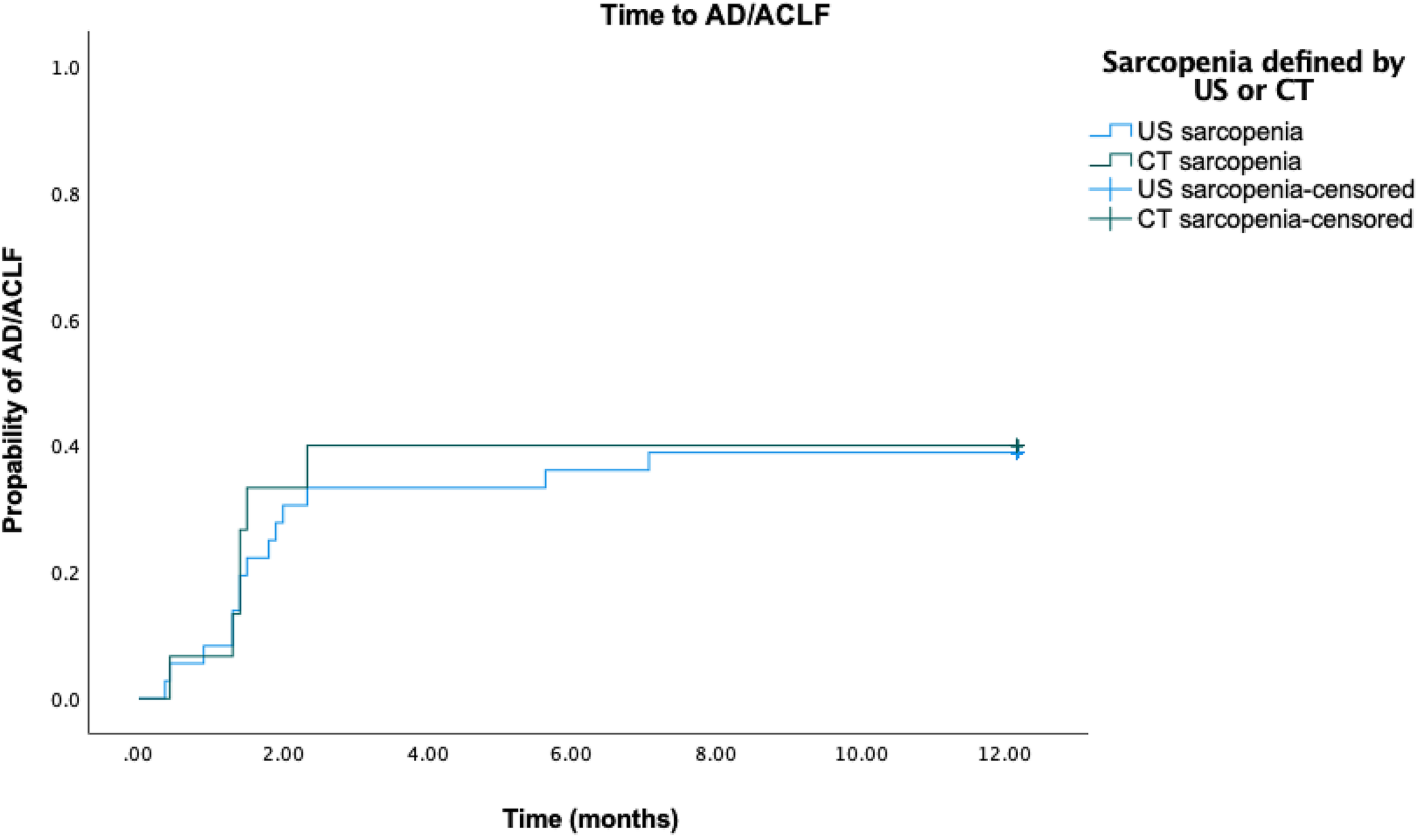
Decompensation of sarcopenic patients defined by CT or US. No significant difference in predicting the outcome can be seen (*p* = 0.899).

US‐ and CT‐defined sarcopenia correlated moderately, Spearman's ρ = 0.423 (*p* = 0.005). Cohen's kappa revealed a moderate but significant agreement between CT and ultrasound defined sarcopenia (*p* = 0.006; κ = 0.379) (Table S4). When stratified for gender, there is only a significant agreement for men (*p* = 0.016), not for women (*p* = 0.118) (Table S5). The occurrence of AD and ACLF did not differ significantly between US‐ and CT‐defined sarcopenia, as demonstrated by the log‐rank test (*p* = 0.899) (Figure 2). Like US‐defined sarcopenia, CT‐defined sarcopenia was also significantly associated with the development of AD within 1 year in univariate analysis (HR 8.7, *p* = 0.01). Besides US defined sarcopenia, presence of CT defined sarcopenia was also independently associated with the development of acute decompensation in multivariate analyses including Child–Pugh (HR7.8, *p* = 0.04). Multivariate analysis with MELD and CT‐defined sarcopenia falls just short of achieving statistical significance but nonetheless delineates a discernible trend (Table 4).

## 4 Discussion

The importance of sarcopenia as a risk factor for liver‐related complications and all‐cause mortality in patients with chronic liver disease has been frequently demonstrated in various studies [5, 6, 7, 9, 24], but there is only limited evidence about US as primary screening modality and its association with the patient outcome in patients with chronic liver disease. Our study suggests that US imaging can serve as an effective diagnostic tool for detecting sarcopenia in liver patients. We observed a notable association between US‐defined sarcopenia and patient outcomes, with non‐sarcopenic patients showing higher overall and 1‐year AD free survival rates. This association appears to be independent of liver function, as measured by MELD and Child–Pugh scores. It is noteworthy that among all patients who were in a controlled status during the initial assessment, only those who were classified as sarcopenic by US decompensated during follow‐up (Figure S2A, Table S2A). Therefore, it can be concluded that not only those who were already in a decompensated status at baseline developed further decompensation more often when sarcopenic, but sarcopenia is also an important risk factor in those who did not suffer from acute decompensation. Those who were already in a decompensated status at baseline developed further decompensation more often when being sarcopenic (Figure S2B and Table S2B).

Most of the studies concerning sarcopenia in cirrhosis have used CT as primary modality to define skeletal muscle index [6, 9, 25]. Importantly, the EASL currently suggests CT‐defined skeletal muscle as the standard technique to assess sarcopenia [16]. However, repetitive CT scans are limited due to radiation exposure, availability and economic reasons, thus leaving an unmet clinical need for a fast and easy‐to‐obtain bedside technique.

Here, we propose to determine the muscle quantity by sonographic measurements of thigh muscle thickness as a cost‐efficient, fast and easy method, which can be used as point‐of‐care diagnostic in sequential examinations in patients with chronic liver disease. Our findings indicate that US might be able both to identify sarcopenia and to monitor treatment response. These results needs to be further validated in prospective studies with greater cohorts, especially to defined sex‐specific US thresholds. US has already been investigated in other studies as a method to evaluate muscle mass and to detect sarcopenia [26, 27, 28]. US measurements in both thigh muscle thickness and psoas muscle thickness have been performed, concluding that US can be used to validly measure muscle size and mass for the detection of sarcopenia. These results align with previous findings of our study group, as the measured US muscle thickness displayed a significant correlation with muscle measurements by CT [29]. Other authors compared different US sites with CT‐derived skeletal muscle index, with quadriceps muscle index to have the strongest correlation [27].

Additionally, we also decided to perform the measurements on the thigh because the muscle loss in the lower extremities is greater than in the upper ones [30]. Differing from our results, it is also suggested by Galindo Martín et al., whose proposed measurement technique we mainly followed, to choose the distal third of the muscle [31] as measurement site. Although we found the proximal third to be the best in our investigations, other measurement locations also showed significant results (*p* < 0.05) in predicting clinical outcome (Table S1).

We were not able to assess interobserver reliability in our cohort due to the limit of repeated examinations but high inter‐ and intraobserver reliability was previously confirmed by various other studies [32, 33].

One of the reasons why US measurements have played little or no role in the detection of sarcopenia is the lack of standardized cut‐off values [34]. We calculated a sarcopenic index as muscle thickness per patient height to ensure coherent and comparable statistical information and then defined an unisex cut‐off value. Although due to limited number of female patients in our study, we were not able to define sex‐specific cut off values. Large population‐based and age‐adjusted studies to define sex specific cut‐offs are needed.

As for the comparability of US and CT, no significant difference in the development of AD and ACLF between the US‐ and CT‐defined sarcopenic group could be seen (Figure 2), with the presence of sarcopenia evaluated by both techniques appearing to increase the risk of AD, respectively (Table 3). Moreover, sarcopenia was revealed to be a relevant risk factor for the development of AD defined both by US and CT (Tables 3 and 4).

We acknowledge limitations to this pilot study. One notable limitation of our study is the wide confidence interval (CI) observed in the measurements of ultrasound‐defined sarcopenia. This large CI indicates a high degree of variability in our data, which can be attributed to several factors. Firstly, ultrasound assessment of muscle mass, while non‐invasive and accessible, is inherently operator dependent. Variations in technique and interpretation among different operators can lead to inconsistencies in measurements.

Moreover, the study population's heterogeneity may have contributed to the variability. Diverse factors such as age, sex, co‐morbidities and lifestyle habits can influence the body composition, leading to broader CIs. For instance, older individuals and those with chronic conditions might exhibit more pronounced sarcopenia, adding to the variability in our sample. Furthermore, skeletal muscle mass is shown to be influenced by overhydration, which can lead to an overassessment of muscle thickness [35].

The wide CI also underscores the need for standardization in ultrasound techniques and the importance of operator training to reduce measurement variability. Future studies should consider these aspects to improve the precision of sarcopenia assessments.

Despite this limitation, our findings provide valuable insights into the prevalence and characteristics of sarcopenia in the studied population. The large CI does not undermine the observed trends but highlights the necessity for caution in interpreting the exact estimates. It calls for more extensive research with standardized protocols and larger sample sizes to confirm our findings and refine the accuracy of ultrasound‐based sarcopenia measurements.

In addition, as already stated, muscle thickness is not the only parameter to determine sarcopenia with certainty. We only assessed muscle mass as parameter for sarcopenia in our study but did not involve functional muscle tests. Geriatric studies revealed a low association between low muscle mass and functionality [36]. A suggestion on an improved identification of patients at risk would be to involve the hand grip strength (HGS). As a parameter measuring muscle strength, HGS has already been shown to be a prognostic marker in patients with listed for liver transplantation [37, 38]. A combination of determination of HGS and our proposed US measurements could potentially identify sarcopenic patients quickly and reliably by simple means.

Lastly, due to the small number of women in our cohort, we refrained from defining sex‐specific US cut‐off values. The unisex US cut‐offs were compared with the sex‐specific cut offs as proposed by the EASL. Therefore, our results might be mainly valid for men. In further studies more precise sex‐specific cut offs should be definied, because sex specific cut‐offs have been displayed to improve the accuracy of the definition of sarcopenia [6, 7, 10, 39]. Moreover, in the sarcopenic group, there were significantly more patients with alcohol‐induced liver cirrhosis, which could be explained due to malnutrition and less physical activity in patients suffering from alcohol misuse.

Our study outlines the applicability of ultrasound in detecting sarcopenia and explores its potential prognostic value in predicting clinical outcomes, particularly the occurrence of acute decompensation and liver‐related mortality. The method demonstrated a relatively high negative predictive value for ascites, suggesting some utility in identifying patients at low risk for future decompensations. Consequently, follow‐up in these patients might be considered for less frequent intervals.

Unlike previous studies that extensively examined the accuracy of ultrasound in identifying sarcopenia compared with CT‐derived approaches, our focus was primarily on a prospective analysis of the influence of ultrasound assessment of thigh muscle mass on clinical outcomes [33, 40].

In summary, our pilot study suggests that ultrasound may be a feasible, easy‐to‐use and efficient method for screening sarcopenia. We observed an association between ultrasound‐defined sarcopenia and a higher prevalence of ascites and overall mortality in patients with chronic liver disease. Future studies should further investigate sonographic imaging as a potential tool to identify patients at risk for ascites and death due to sarcopenia and to assess sarcopenia in follow‐up examinations.

## Author Contributions

J.G. and L.S.: acquisition of data, analysis and interpretation of data, drafting of the manuscript, statistical analysis. T.J., S.N.R.G., N.B., M.K., J.A.M., K.H.P., and J.T.: interpretation of data, critical revision of the manuscript regarding important intellectual content. J.C. and M.P.: study concept and design, analysis and interpretation of data, drafting of the manuscript, critical revision of the manuscript regarding important intellectual content, final approval of the version to be published, administrative, technical and material support, study supervision.

## Ethics Statement

This study was conducted according to the principles in the Declaration of Helsinki and was approved by the local ethics committee (ethical vote number 095/16, University Bonn). All patients gave informed consent. The study was registered in the public repository ClinicalTrials.gov under the identifier NCT03584204.

## Conflicts of Interest

The authors declare no conflicts of interest.

## Supporting information


**Figure S1.** A. AD/ACLF‐free survival of the subgroup of compensated patients at baseline.
**Figure S2.** B. AD/ACLF‐free survival of the subgroup of decompensated patients at baseline.
**Table S1.** A comparative analysis of the various measuring sights of the upper thigh utilized in ultrasound, with and without pressure performed by Analysis of variance (ANOVA).
**Table S2.** A. Number of acute decompensation events stratified US‐defined sarcopenia by US‐SMI in compensated patents at baseline. B. Number of acute decompensation events stratified US‐defined sarcopenia by US‐SMI in decompensated patents at baseline.
**Table S3.** Number of acute decompensation events stratified by US‐defined sarcopenia by US‐SMI.
**Table S4.** Agreement of categorization by CT‐defined and US‐defined sarcopenia by Cohens kappa.
**Table S5.** Agreement of categorization by CT‐defined and US‐defined sarcopenia by Cohens kappa stratified by sex. Abbreviations: ACLF = acute‐on‐chronic liver failure; AD = acute decompensation; BL = baseline; US‐SMI = ultrasound‐defined skeletal muscle index.
**Table S6.** STROBE Checklist.

## References

[1] A. J. Cruz‐Jentoft , J. P. Baeyens , J. M. Bauer , et al., “Sarcopenia: European Consensus on Definition and Diagnosis,” Age and Ageing 39, no. 4 (2010 Jul): 412–423. 20392703 10.1093/ageing/afq034 PMC2886201

[2] J. Traub , I. Bergheim , M. Eibisberger , and V. Stadlbauer , “Sarcopenia and Liver Cirrhosis—Comparison of the European Working Group on Sarcopenia Criteria 2010 and 2019,” Nutrients 12, no. 2 (2020): 547. 32093198 10.3390/nu12020547 PMC7071440

[3] T. Hanai , M. Shiraki , K. Nishimura , et al., “Sarcopenia Impairs Prognosis of Patients With Liver Cirrhosis,” Nutrition 31, no. 1 (2015): 193–199. 25441595 10.1016/j.nut.2014.07.005

[4] H. Y. Kim and J. W. Jang , “Sarcopenia in the Prognosis of Cirrhosis: Going Beyond the MELD Score,” World Journal of Gastroenterology 21, no. 25 (2015): 7637–7647. 26167066 10.3748/wjg.v21.i25.7637 PMC4491953

[5] X. Tantai , Y. Liu , Y. H. Yeo , et al., “Effect of Sarcopenia on Survival in Patients With Cirrhosis: A Meta‐Analysis,” Journal of Hepatology 76, no. 3 (2022): 588–599. 34785325 10.1016/j.jhep.2021.11.006

[6] M. Praktiknjo , C. Clees , A. Pigliacelli , et al., “Sarcopenia Is Associated With Development of Acute‐on‐Chronic Liver Failure in Decompensated Liver Cirrhosis Receiving Transjugular Intrahepatic Portosystemic Shunt,” Clinical and Translational Gastroenterology 10, no. 4 (2019): e00025. 30939488 10.14309/ctg.0000000000000025 PMC6602782

[7] M. Praktiknjo , M. Book , J. Luetkens , et al., “Fat‐Free Muscle Mass in Magnetic Resonance Imaging Predicts Acute‐on‐Chronic Liver Failure and Survival in Decompensated Cirrhosis,” Hepatology 67, no. 3 (2018): 1014–1026. 29059469 10.1002/hep.29602

[8] A. Faron , J. Abu‐Omar , J. Chang , N. Böhling , et al., “Combination of Fat‐Free Muscle Index and Total Spontaneous Portosystemic Shunt Area Identifies High‐Risk Cirrhosis Patients,” Frontiers in Medicine 9 (2022): 9, 10.3389/fmed.2022.831005. PMC9040492 35492329

[9] E. J. Carey , J. C. Lai , C. W. Wang , et al., “Fitness, Life Enhancement, and Exercise in Liver Transplantation Consortium a Multicenter Study to Define Sarcopenia in Patients With End‐Stage Liver Disease,” Liver Transplantation 23, no. 5 (2017): 625–633. 28240805 10.1002/lt.24750 PMC5762612

[10] N. Golse , P. O. Bucur , O. Ciacio , et al., “A new Definition of Sarcopenia in Patients With Cirrhosis Undergoing Liver Transplantation,” Liver Transplantation 23, no. 2 (2017): 143–154. 28061014 10.1002/lt.24671

[11] G. Davuluri , A. Allawy , S. Thapaliya , et al., “Hyperammonaemia‐Induced Skeletal Muscle Mitochondrial Dysfunction Results in Cataplerosis and Oxidative Stress,” Journal of Physiology 594, no. 24 (2016): 7341–7360. 27558544 10.1113/JP272796 PMC5157075

[12] J. McDaniel , G. Davuluri , E. A. Hill , et al., “Hyperammonemia Results in Reduced Muscle Function Independent of Muscle Mass,” American Journal of Physiology. Gastrointestinal and Liver Physiology 310, no. 3 (2016): G163–G170. 26635319 10.1152/ajpgi.00322.2015 PMC4971815

[13] S. Dasarathy and M. Merli , “Sarcopenia From Mechanism to Diagnosis and Treatment in Liver Disease,” Journal of Hepatology 65, no. 6 (2016): 1232–1244. 27515775 10.1016/j.jhep.2016.07.040 PMC5116259

[14] J. Qiu , S. Thapaliya , A. Runkana , et al., “Hyperammonemia in Cirrhosis Induces Transcriptional Regulation of Myostatin by an NF‐κB–Mediated Mechanism,” Proceedings of the National Academy of Sciences of the United States of America 110, no. 45 (2013): 18162–18167. 24145431 10.1073/pnas.1317049110 PMC3831479

[15] J. Qiu , C. Tsien , S. Thapalaya , et al., “Hyperammonemia‐Mediated Autophagy in Skeletal Muscle Contributes to Sarcopenia of Cirrhosis,” American Journal of Physiology. Endocrinology and Metabolism 303, no. 8 (2012): E983–E993. 22895779 10.1152/ajpendo.00183.2012 PMC3469607

[16] M. Merli , A. Berzigotti , S. Zelber‐Sagi , et al., “EASL Clinical Practice Guidelines on Nutrition in Chronic Liver Disease,” Journal of Hepatology 70, no. 1 (2019): 172–193. 30144956 10.1016/j.jhep.2018.06.024 PMC6657019

[17] P. R. Galle , A. Forner , J. M. Llovet , et al., “EASL Clinical Practice Guidelines: Management of Hepatocellular Carcinoma,” Journal of Hepatology 69, no. 1 (2018): 182–236. 29628281 10.1016/j.jhep.2018.03.019

[18] A. Ticinesi , T. Meschi , M. V. Narici , F. Lauretani , and M. Maggio , “Muscle Ultrasound and Sarcopenia in Older Individuals: A Clinical Perspective,” Journal of the American Medical Directors Association 18, no. 4 (2017): 290–300. 28202349 10.1016/j.jamda.2016.11.013

[19] M. A. Minetto , C. Caresio , T. Menapace , et al., “Ultrasound‐Based Detection of Low Muscle Mass for Diagnosis of Sarcopenia in Older Adults,” Pm&r 8, no. 5 (2016): 453–462. 26431809 10.1016/j.pmrj.2015.09.014

[20] M. Mourtzakis , S. Parry , B. Connolly , and Z. Puthucheary , “Skeletal Muscle Ultrasound in Critical Care: A Tool in Need of Translation,” Annals of the American Thoracic Society 14, no. 10 (2017): 1495–1503. 28820608 10.1513/AnnalsATS.201612-967PS PMC5718569

[21] R. de Franchis , J. Bosch , G. Garcia‐Tsao , et al., “Baveno VII – Renewing Consensus in Portal Hypertension,” Journal of Hepatology 76, no. 4 (2022): 959–974. 35120736 10.1016/j.jhep.2021.12.022 PMC11090185

[22] R. Moreau , R. Jalan , P. Gines , et al., “Acute‐on‐Chronic Liver Failure Is a Distinct Syndrome That Develops in Patients With Acute Decompensation of Cirrhosis,” Gastroenterology 144, no. 7 (2013): 1426–1437. 23474284 10.1053/j.gastro.2013.02.042

[23] P. Tandon , A. J. Montano‐Loza , J. C. Lai , S. Dasarathy , and M. Merli , “Sarcopenia and Frailty in Decompensated Cirrhosis,” Journal of Hepatology 75, no. Suppl 1 (2021 Jul): S147–S162. 34039486 10.1016/j.jhep.2021.01.025 PMC9125684

[24] S. Nardelli , B. Lattanzi , M. Merli , et al., “Muscle Alterations Are Associated With Minimal and Overt Hepatic Encephalopathy in Patients With Liver Cirrhosis,” Hepatology 70, no. 5 (2019): 1704–1713. 31038758 10.1002/hep.30692

[25] F. Durand , S. Buyse , C. Francoz , et al., “Prognostic Value of Muscle Atrophy in Cirrhosis Using Psoas Muscle Thickness on Computed Tomography,” Journal of Hepatology 60, no. 6 (2014): 1151–1157. 24607622 10.1016/j.jhep.2014.02.026

[26] P. Tandon , M. Ney , I. Irwin , et al., “Severe Muscle Depletion in Patients on the Liver Transplant Wait List: Its Prevalence and Independent Prognostic Value,” Liver Transplantation 18, no. 10 (2012): 1209–1216. 22740290 10.1002/lt.23495

[27] S. Dhariwal , A. Roy , S. Taneja , et al., “Assessment of Sarcopenia Using Muscle Ultrasound in Patients With Cirrhosis and Sarcopenic Obesity (AMUSE STUDY),” Journal of Clinical Gastroenterology 9 (2022): 841–847. 10.1097/MCG.0000000000001745 35943413

[28] A. Hari , A. Berzigotti , B. Štabuc , and N. Caglevič , “Muscle Psoas Indices Measured by Ultrasound in Cirrhosis ‐ Preliminary Evaluation of Sarcopenia Assessment and Prediction of Liver Decompensation and Mortality,” Digestive and Liver Disease 51, no. 11 (2019): 1502–1507. 31547952 10.1016/j.dld.2019.08.017

[29] J. Gödiker , K. Krüger , L. Schwind , et al., “Letter: The Diagnostic Value of Ultrasound‐Based Versus CT‐Based Sarcopenia Measurement in Cirrhosis,” Alimentary Pharmacology & Therapeutics 57, no. 5 (2023): 591–592. 36786459 10.1111/apt.17343

[30] P. Turton , R. Hay , J. Taylor , J. McPhee , and I. Welters , “Human Limb Skeletal Muscle Wasting and Architectural Remodeling During Five to Ten Days Intubation and Ventilation in Critical Care – An Observational Study Using Ultrasound,” BMC Anesthesiology 29, no. 16 (2016): 119. 10.1186/s12871-016-0269-z PMC5127036 27894277

[31] C. A. Galindo Martín , E. Monares Zepeda , and O. A. Lescas Méndez , “Bedside Ultrasound Measurement of Rectus Femoris: A Tutorial for the Nutrition Support Clinician,” Journal of Nutrition and Metabolism 2017 (2017): 2767232. 28386479 10.1155/2017/2767232 PMC5366786

[32] M. Tillquist , D. J. Kutsogiannis , P. E. Wischmeyer , et al., “Bedside Ultrasound Is a Practical and Reliable Measurement Tool for Assessing Quadriceps Muscle Layer Thickness,” JPEN Journal of Parenteral and Enteral Nutrition 38, no. 7 (2014): 886–890. 23980134 10.1177/0148607113501327 PMC4502435

[33] P. Tandon , G. Low , M. Mourtzakis , et al., “A Model to Identify Sarcopenia in Patients With Cirrhosis,” Clinical Gastroenterology and Hepatology 14, no. 10 (2016): 1473–1480.e3. 27189915 10.1016/j.cgh.2016.04.040

[34] D. Albano , C. Messina , J. Vitale , and L. M. Sconfienza , “Imaging of Sarcopenia: Old Evidence and New Insights,” European Radiology 30, no. 4 (2020): 2199–2208. 31834509 10.1007/s00330-019-06573-2

[35] C. I. Wells , J. L. McCall , and L. D. Plank , “Relationship Between Total Body Protein and Cross‐Sectional Skeletal Muscle Area in Liver Cirrhosis Is Influenced by Overhydration,” Liver Transplantation 25, no. 1 (2019): 45–55. 30040184 10.1002/lt.25314

[36] L. A. Schaap , A. Koster , and M. Visser , “Adiposity, Muscle Mass, and Muscle Strength in Relation to Functional Decline in Older Persons,” Epidemiologic Reviews 35, no. 1 (2013): 51–65. 23221972 10.1093/epirev/mxs006

[37] M. Sinclair , B. Chapman , R. Hoermann , et al., “Handgrip Strength Adds More Prognostic Value to the Model for End‐Stage Liver Disease Score Than Imaging‐Based Measures of Muscle Mass in Men With Cirrhosis,” Liver Transplantation 25, no. 10 (2019): 1480–1487. 31282126 10.1002/lt.25598

[38] C. W. Wang , S. Feng , K. E. Covinsky , et al., “A Comparison of Muscle Function, Mass, and Quality in Liver Transplant Candidates: Results From the Functional Assessment in Liver Transplantation (FrAILT) Study,” Transplantation 100, no. 8 (2016): 1692–1698. 27314169 10.1097/TP.0000000000001232 PMC4962324

[39] A. J. Montano‐Loza , J. Meza‐Junco , C. M. M. Prado , et al., “Muscle Wasting Is Associated With Mortality in Patients With Cirrhosis,” Clinical Gastroenterology and Hepatology 10, no. 2 (2012): 166–173.e1. 21893129 10.1016/j.cgh.2011.08.028

[40] S. Dhariwal , A. Roy , S. Taneja , et al., “Assessment of Sarcopenia Using Muscle Ultrasound in Patients With Cirrhosis and Sarcopenic Obesity (AMUSE STUDY),” Journal of Clinical Gastroenterology 57, no. 8 (2023): 841–847. 35943413 10.1097/MCG.0000000000001745

