# Supplementary material for: Ultrasound‐Defined Sarcopenia Independently Predicts Acute Decompensation in Advanced Chronic Liver Disease
Source: J Cachexia Sarcopenia Muscle. 2024 Nov 11;15(6):2792–802. doi: 10.1002/jcsm.13630 (PMC11634521; doi:10.1002/jcsm.13630)
Supplement: Supplementary file 1 — Figure S1. A. AD/ACLF‐free survival of the subgroup of compensated patients at baseline. Figure S2. B. AD/ACLF‐free survival of the subgroup of decompensated patients at baseline. Table S1. A comparative analysis of the various measuring sights of the upper thigh utilized in ultrasound, with and without pressure performed by Analysis of variance (ANOVA). Table S2. A. Number of acute decompensation events stratified US‐defined sarcopenia by US‐SMI in compensated patents at baseline. B. Number of acute decompensation events stratified US‐defined sarcopenia by US‐SMI in decompensated patents at baseline. Table S3. Number of acute decompensation events stratified by US‐defined sarcopenia by US‐SMI. Table S4. Agreement of categorization by CT‐defined and US‐defined sarcopenia by Cohens kappa. Table S5. Agreement of categorization by CT‐defined and US‐defined sarcopenia by Cohens kappa stratified by sex. Abbreviations: ACLF = acute‐on‐chronic liver failure; AD = acute decompensation; BL = baseline; US‐SMI = ultrasound‐defined skeletal muscle index. Table S6. STROBE Checklist. [file JCSM-15-2792-s001.docx]

**SUPPLEMENTARY MATERIALS**

**Ultrasound-defined sarcopenia independently predicts acute decompensation in advanced chronic liver disease**

**Table of Content**

**Supplementary Figure 1.** Patient cohort flow chart.

**Supplementary Figure 1A.** AD/ACLF-free survival of the subgroup of compensated patients at baseline.

**Supplementary Figure 2B.** AD/ACLF-free survival of the subgroup of decompensated patients at baseline.

**Supplementary Table 1**. A comparative analysis of the various measuring sights of the upper thigh utilized in ultrasound, with and without pressure performed by Analysis of variance (ANOVA).

**Supplementary Table 2A.** Number of acute decompensation events stratified US-defined sarcopenia by US-SMI in compensated patents at baseline.

**Supplementary Table 2B.** Number of acute decompensation events stratified US-defined sarcopenia by US-SMI in decompensated patents at baseline.

**Supplementary Table 3.** Number of acute decompensation events stratified by US-defined sarcopenia by US-SMI.

**Supplementary Table 4.** Agreement of categorization by CT-defined and US-defined sarcopenia by Cohens kappa.

**Supplementary Table 5.** Agreement of categorization by CT-defined and US-defined sarcopenia by Cohens kappa stratified by sex. Abbreviations: ACLF=acute-on-chronic liver failure; AD=acute decompensation; BL=baseline; US-SMI=ultrasound- defined skeletal muscle index.

**Supplementary Table 6.** STROBE Checklist.

**Supplementary Figure 1**. Patient cohort flow chart.

**Supplementary Figure 2A.** AD/ACLF-free survival of the subgroup of compensated patients at baseline. Sarcopenic patients showed significantly higher rates of AD/ACLF than non-sarcopenic patients. P by log-rank = 0.001.


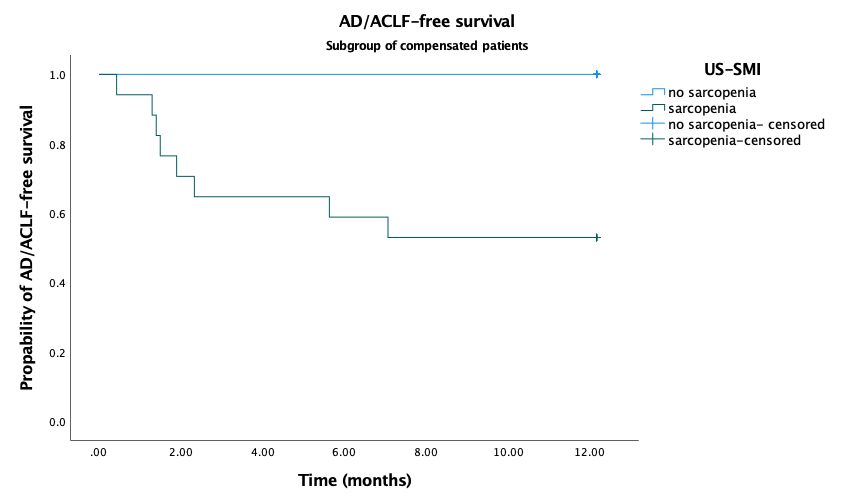


**Supplementary Figure 2B.** AD/ACLF-free survival of the subgroup of decompensated patients at baseline. Sarcopenic patients showed numerically higher rates of AD/ACLF compared to non-sarcopenic patients. This difference was not statistically significant. P by log-rank =0.449.


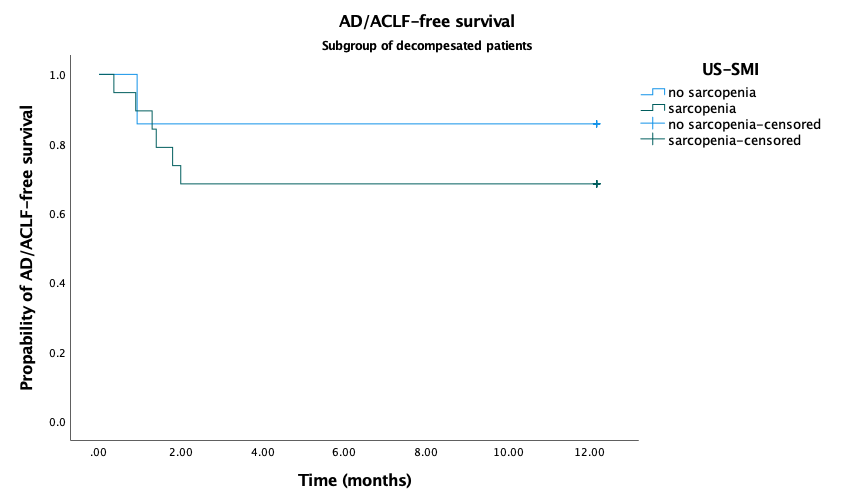


**Supplementary Table 1**. A comparative analysis of the various measuring sights of the upper thigh utilized in ultrasound, with and without pressure performed by Analysis of variance (ANOVA).

|  | | Sum of Squares | df | Mean Square | F | Sig. |
| --- | --- | --- | --- | --- | --- | --- |
| Femoral muscle thickness proximal third left no pressure | Between Groups | 90553.970 | 1 | 90553.970 | 7.820 | **.007** |
|  | Within Groups | 706353.324 | 61 | 11579.563 |  |  |
|  | Total | 796907.295 | 62 |  |  |  |
| Femoral muscle thickness proximal third left with pressure | Between Groups | 25920.851 | 1 | 25920.851 | 5.744 | **.020** |
|  | Within Groups | 275272.833 | 61 | 4512.669 |  |  |
|  | Total | 301193.685 | 62 |  |  |  |
| Femoral muscle thickness middle third left no pressure | Between Groups | 29219.507 | 1 | 29219.507 | 4.889 | **.031** |
|  | Within Groups | 364578.169 | 61 | 5976.691 |  |  |
|  | Total | 393797.675 | 62 |  |  |  |
| Femoral muscle thickness middle third left with pressure | Between Groups | 9450.993 | 1 | 9450.993 | 3.290 | .075 |
|  | Within Groups | 175233.350 | 61 | 2872.678 |  |  |
|  | Total | 184684.342 | 62 |  |  |  |
| Femoral muscle thickness distal third left no pressure | Between Groups | 13920.114 | 1 | 13920.114 | 4.334 | **.042** |
|  | Within Groups | 195916.044 | 61 | 3211.738 |  |  |
|  | Total | 209836.159 | 62 |  |  |  |
| Femoral muscle thickness distal third left with pressure | Between Groups | 3062.905 | 1 | 3062.905 | 1.842 | .180 |
|  | Within Groups | 101404.968 | 61 | 1662.377 |  |  |
|  | Total | 104467.873 | 62 |  |  |  |
| Femoral muscle thickness proximal third right no pressure | Between Groups | 68306.207 | 1 | 68306.207 | 8.004 | **.006** |
|  | Within Groups | 520602.135 | 61 | 8534.461 |  |  |
|  | Total | 588908.342 | 62 |  |  |  |
| Femoral muscle thickness proximal third right with pressure | Between Groups | 25186.067 | 1 | 25186.067 | 4.509 | **.038** |
|  | Within Groups | 340720.370 | 61 | 5585.580 |  |  |
|  | Total | 365906.437 | 62 |  |  |  |
| Femoral muscle thickness middle third right no pressure | Between Groups | 29814.829 | 1 | 29814.829 | 5.085 | **.028** |
|  | Within Groups | 357688.498 | 61 | 5863.746 |  |  |
|  | Total | 387503.326 | 62 |  |  |  |
| Femoral muscle thickness middle third right with pressure | Between Groups | 8641.111 | 1 | 8641.111 | 3.061 | .085 |
|  | Within Groups | 172222.183 | 61 | 2823.314 |  |  |
|  | Total | 180863.295 | 62 |  |  |  |
| Femoral muscle thickness distal third right no pressure | Between Groups | 9382.648 | 1 | 9382.648 | 3.152 | .081 |
|  | Within Groups | 181602.509 | 61 | 2977.090 |  |  |
|  | Total | 190985.157 | 62 |  |  |  |
| Femoral muscle thickness distal third right with pressure | Between Groups | 4333.356 | 1 | 4333.356 | 2.483 | .120 |
|  | Within Groups | 104703.863 | 60 | 1745.064 |  |  |
|  | Total | 109037.219 | 61 |  |  |  |

**Supplementary Table 2A.** Number of acute decompensation events stratified US-defined sarcopenia by US-SMI in compensated patents at baseline. Abbreviations: ACLF=acute-on-chronic liver failure; AD=acute decompensation; BL=baseline; US-SMI=ultrasound- defined skeletal muscle index.

| Decompensation status at BL | | | | US-SMI | | Total |
| --- | --- | --- | --- | --- | --- | --- |
|  |  |  |  | non-sarcopenic | sarcopenic |  |
| compensated | primary endpoint (AD/ACLF) | no AD | count | 20 | 9 | 29 |
|  |  |  | % of AD/ACLF | 69.0% | 31.0% | 100.0% |
|  |  |  | % of US-SMI | 100.0% | 52.9% | 78.4% |
|  |  | AD | count | 0 | 8 | 8 |
|  |  |  | % of AD/ACLF | 0.0% | 100.0% | 100.0% |
|  |  |  | % of US-SMI | 0.0% | 47.1% | 21.6% |
|  | Total | | count | 20 | 17 | 37 |
|  |  |  | % of AD/ACLF | 54.1% | 45.9% | 100.0% |
|  |  |  | % of US-SMI | 100.0% | 100.0% | 100.0% |

**Supplementary Table 2B.** Number of acute decompensation events stratified US-defined sarcopenia by US-SMI in decompensated patents at baseline. ACLF=acute-on-chronic liver failure; AD=acute decompensation; BL=baseline; US-SMI=ultrasound- defined skeletal muscle index.

| Decompensation status at BL | | | | US-SMI | | Total |
| --- | --- | --- | --- | --- | --- | --- |
|  |  |  |  | non-sarcopenic | sarcopenic |  |
| decompensated | primary endpoint (AD/ACLF) | no AD | count | 6 | 13 | 19 |
|  |  |  | % of AD/ACLF | 31.6% | 68.4% | 100.0% |
|  |  |  | % of US-SMI | 85.7% | 68.4% | 73.1% |
|  |  | AD | count | 1 | 6 | 7 |
|  |  |  | % of AD/ACLF | 14.3% | 85.7% | 100.0% |
|  |  |  | % of US-SMI | 14.3% | 31.6% | 26.9% |
|  | Total | | count | 7 | 19 | 26 |
|  |  |  | % of AD/ACLF | 26.9% | 73.1% | 100.0% |
|  |  |  | % of US-SMI | 100.0% | 100.0% | 100.0% |

**Supplementary Table 3.** Number of acute decompensation events stratified by US-defined sarcopenia by US-SMI. Abbreviations: ACLF=acute-on-chronic liver failure; AD=acute decompensation; BL=baseline; US-SMI=ultrasound- defined skeletal muscle index.

|  | | | US-SMI | | Total |
| --- | --- | --- | --- | --- | --- |
|  |  |  | no sarcopenia | sarcopenia |  |
| AD status | no AD/ACLF | Count | 26 | 22 | 48 |
|  |  | % of US-SMI | 96.3% | 61.1% | 76.0% |
|  | AD/ACLF | Count | 1 | 14 | 15 |
|  |  | % of US-SMI | 3.7% | 38.9% | 23.8% |
| Total | | Count | 27 | 36 | 63 |
|  |  | % of US-SMI | 100.0% | 100.0% | 100.0% |

**Supplementary Table 4.** Agreement of categorization by CT-defined and US-defined sarcopenia by Cohens kappa. Abbreviations: ACLF=acute-on-chronic liver failure; AD=acute decompensation; BL=baseline; US-SMI=ultrasound- defined skeletal muscle index.

|  | | | CT-SMI | | Total |
| --- | --- | --- | --- | --- | --- |
|  |  |  | non-sarcopenic | sarcopenic |  |
| US-SMI | non-sarcopenic | Count | 16 | 2 | 18 |
|  |  | % of CT-SMI | 57.1% | 13.3% | 41.9% |
|  | sarcopenic | Count | 12 | 13 | 25 |
|  |  | % of CT-SMI | 42.9% | 86.7% | 58.1% |
| Total | | Count | 28 | 15 | 43 |
|  |  | % of CT-SMI | 100.0% | 100.0% | 100.0% |

|  | | Value | Asymptotic Standard Error^a^ | Approximate t | Approximate Significance |
| --- | --- | --- | --- | --- | --- |
| Measure of Agreement | Kappa | .379 | .123 | 2.775 | .006 |
| Number of valid cases | | 43 |  |  |  |
|  | | | | | |
|  | | | | | |

**Supplementary Table 5.** Agreement of categorization by CT-defined and US-defined sarcopenia by Cohens kappa stratified by sex. Abbreviations: ACLF=acute-on-chronic liver failure; AD=acute decompensation; BL=baseline; US-SMI=ultrasound- defined skeletal muscle index.

| sex | | | | | | | CT-SMI | | | | Total |
| --- | --- | --- | --- | --- | --- | --- | --- | --- | --- | --- | --- |
|  |  |  |  |  |  |  | non-sarcopenic | | sarcopenic | |  |
| male | US-SMI | | non-sarcopenic | | Count | | 11 | | 2 | | 13 |
|  |  |  |  |  | % of CT-SMI | | 61.1% | | 16.7% | | 43.3% |
|  |  |  | sarcopenic | | Count | | 7 | | 10 | | 17 |
|  |  |  |  |  | % of CT-SMI | | 38.9% | | 83.3% | | 56.7% |
|  | Total | | | | Count | | 18 | | 12 | | 30 |
|  |  |  |  |  | % of CT-SMI | | 100.0% | | 100.0% | | 100.0% |
| female | US-SMI | | non-sarcopenic | | Count | | 5 | | 0 | | 5 |
|  |  |  |  |  | % of CT-SMI | | 50.0% | | 0.0% | | 38.5% |
|  |  |  | sarcopenic | | Count | | 5 | | 3 | | 8 |
|  |  |  |  |  | % of CT-SMI | | 50.0% | | 100.0% | | 61.5% |
|  | Total | | | | Count | | 10 | | 3 | | 13 |
|  |  |  |  |  | % of CT-SMI | | 100.0% | | 100.0% | | 100.0% |
|  | | | | | | | | | | | |
| Sex | | | | | | Value | Asymptotic Standard Error r^a^ | Approximate t | | Approximate significance | |
| male | | Measure of Agreement | | Kappa | | .416 | .155 | 2.407 | | .016 | |
|  |  | Number of valid cases | | | | 30 |  |  | |  | |
| female | | Measure of Agreement | | Kappa | | .316 | .175 | 1.561 | | .118 | |
|  |  | Number of valid cases | | | | 13 |  |  | |  | |

**Supplementary Table 6.** STROBE Checklist.

|  | Item No | Recommendation | Ultrasound Sarcopenia in chronic liver disease |  |  |  |
| --- | --- | --- | --- | --- | --- | --- |
| **Title and abstract** | 1 | (*a*) Indicate the study’s design with a commonly used term in the title or the abstract | p. 2, l. 37 |  |  |  |
|  |  | (*b*) Provide in the abstract an informative and balanced summary of what was done and what was found | pp. 2-3, ll. 26-61 |  |  |  |
| Introduction | | |  |  |  |  |
| Background/rationale | 2 | Explain the scientific background and rationale for the investigation being reported | pp. 3-5, ll. 72-115 |  |  |  |
| Objectives | 3 | State specific objectives, including any prespecified hypotheses | p. 5, ll. 116-118 |  |  |  |
| Methods | | |  |  |  |  |
| Study design | 4 | Present key elements of study design early in the paper | pp. 5-6., ll. 122-132 |  |  |  |
| Setting | 5 | Describe the setting, locations, and relevant dates, including periods of recruitment, exposure, follow-up, and data collection | p. 5, ll.122-124 |  |  |  |
| Participants | 6 | (*a*) Give the eligibility criteria, and the sources and methods of selection of participants. Describe methods of follow-up | pp. 5-6, ll. 122-132 |  |  |  |
|  |  | (*b*) For matched studies, give matching criteria and number of exposed and unexposed | NA |  |  |  |
| Variables | 7 | Clearly define all outcomes, exposures, predictors, potential confounders, and effect modifiers. Give diagnostic criteria, if applicable | pp. 5-8, ll. 120-176 |  |  |  |
| Data sources/ measurement | 8* | For each variable of interest, give sources of data and details of methods of assessment (measurement). Describe comparability of assessment methods if there is more than one group | pp. 5-8, ll. 120-176 |  |  |  |
| Bias | 9 | Describe any efforts to address potential sources of bias | pp. 5-8, ll. 120-176 |  |  |  |
| Study size | 10 | Explain how the study size was arrived at | pp. 5-6, ll. 122-126 |  |  |  |
| Quantitative variables | 11 | Explain how quantitative variables were handled in the analyses. If applicable, describe which groupings were chosen and why | pp. 5-8, ll. 120-176 |  |  |  |
| Statistical methods | 12 | (*a*) Describe all statistical methods, including those used to control for confounding | pp. 7-8, ll. 157-176 |  |  |  |
|  |  | (*b*) Describe any methods used to examine subgroups and interactions | pp. 7-8, ll. 157-176 |  |  |  |
|  |  | (*c*) Explain how missing data were addressed | pp. 7-8, ll. 157-176 |  |  |  |
|  |  | (*d*) If applicable, explain how loss to follow-up was addressed | pp. 7-8, ll. 157-176 |  |  |  |
|  |  | (*e*) Describe any sensitivity analyses | NA |  |  |  |
| Results | | |  |  |  |  |
| Participants | 13* | (a) Report numbers of individuals at each stage of study—eg numbers potentially eligible, examined for eligibility, confirmed eligible, included in the study, completing follow-up, and analysed | See flow chart. Supplementary Figure 1. |  |  |  |
|  |  | (b) Give reasons for non-participation at each stage | See flow chart. Supplementary Figure 1. |  |  |  |
|  |  | (c) Consider use of a flow diagram | See flow chart. Supplementary Figure 1. |  |  |  |
| Descriptive data | 14* | (a) Give characteristics of study participants (eg demographic, clinical, social) and information on exposures and potential confounders | p. 8, ll. 179-192 |  |  |  |
|  |  | (b) Indicate number of participants with missing data for each variable of interest | p. 8, ll. 179-192 |  |  |  |
|  |  | (c) Summarise follow-up time (eg, average and total amount) | p. 8, ll. 179-192 |  |  |  |
| Outcome data | 15* | Report numbers of outcome events or summary measures over time | pp. 8-12, ll. 194-285 |  |  |  |
| Main results | 16 | (*a*) Give unadjusted estimates and, if applicable, confounder-adjusted estimates and their precision (eg, 95% confidence interval). Make clear which confounders were adjusted for and why they were included | pp. 8-12, ll. 194-285 |  |  |  |
|  |  | (*b*) Report category boundaries when continuous variables were categorized | NA |  |  |  |
|  |  | (*c*) If relevant, consider translating estimates of relative risk into absolute risk for a meaningful time period | NA |  |  |  |
| Other analyses | 17 | Report other analyses done—eg analyses of subgroups and interactions, and sensitivity analyses | pp. 8-12, ll. 194-285 |  |  |  |
| Discussion | | |  |  |  |  |
| Key results | 18 | Summarise key results with reference to study objectives | pp. 12-13, ll.287-305 |  |  |  |
| Limitations | 19 | Discuss limitations of the study, taking into account sources of potential bias or imprecision. Discuss both direction and magnitude of any potential bias | pp. 15-16, ll.350-392 |  |  |  |
| Interpretation | 20 | Give a cautious overall interpretation of results considering objectives, limitations, multiplicity of analyses, results from similar studies, and other relevant evidence | pp. 16-17, ll. 393-409 |  |  |  |
| Generalisability | 21 | Discuss the generalisability (external validity) of the study results | pp. 16-17, ll. 393-409 |  |  |  |
| Other information | | |  |  |  |  |
| Funding | 22 | Give the source of funding and the role of the funders for the present study and, if applicable, for the original study on which the present article is based | pp. 17-18, ll. 420-428 |  |  |  |
